# Can decreased femoral head enhancement differentiate between septic hip arthritis and transient synovitis?

**DOI:** 10.1007/s00256-025-05013-1

**Published:** 2025-08-12

**Authors:** Boaz Karmazyn, Christopher L. Newman, Monica M. Forbes-Amrhein, Andrea M. E. Palazzolo Ray, Willa R. Schmahl, S. Gregory Jennings, George J. Eckert, Erika L. Daley

**Affiliations:** 1 Department of Radiology and Imaging Sciences, Riley Hospital for Children at IU Health, Indiana University School of Medicine, 705 Riley Hospital Dr., Indianapolis, IN 46202 USA; 2 Department of Orthopaedic Surgery, Indiana University Hospital, Indiana University Health University Hospital, Indiana University School of Medicine, 550 N. University Blvd., Suite 6201, Indianapolis, IN 46202 USA; 3 Department of Orthopaedic Surgery, Feinberg School of Medicine, Northwestern University, 259 E Erie St., Chicago, IL 60611 USA; 4 Department of Radiology and Imaging Sciences, Indiana University School of Medicine, 950 W. Walnut Street Rm E124, Indianapolis, IN 46202 USA; 5 Department of Biostatistics and Health Data Science, Indiana University School of Medicine, 340 West 10th Street Fairbanks Hall, Indianapolis, IN 46202 USA; 6 Department of Pediatric Orthopedics and Sports Medicine, Riley Hospital for Children at IU Health, Indiana University School of Medicine, 575 Riley Hospital Dr., Indianapolis, IN 46202 USA

**Keywords:** Septic arthritis, Transient synovitis, MRI, Decreased enhancement

## Abstract

**Objective:**

To determine whether decreased femoral head enhancement on MRI differentiates septic arthritis from transient synovitis.

**Materials and methods:**

This retrospective study included children < 10 years old with hip effusion on post-contrast MRI for suspected musculoskeletal infection. Two pediatric radiologists independently assessed femoral head enhancement. Kocher and modified Kocher scores were calculated from clinical and lab data. Differences between septic arthritis and transient synovitis were analyzed using Student’s *t*-tests and Fisher’s exact tests. Sensitivity and specificity for diagnosing septic arthritis were calculated for Kocher scores, their individual components, decreased femoral head enhancement, and muscle edema. Interobserver agreement was assessed.

**Results:**

Thirty-four children were included (20 transient synovitis, 14 septic arthritis). Kocher and modified Kocher scores were significantly higher in septic arthritis (*p* = 0.003, 0.008). Interobserver agreement for femoral head enhancement was substantial (kappa = 0.70). On consensus read, decreased femoral head enhancement was seen in 71.4% of septic arthritis and 50.0% of transient synovitis cases (*p* = 0.296). Bone marrow edema was present in two septic arthritis cases. Muscle edema had moderate to high sensitivity (71.4%, 92.9%) but moderate to low specificity (75.0%, 50.0%) for septic arthritis.

**Conclusion:**

Decreased femoral head enhancement does not reliably distinguish septic arthritis from transient synovitis. Relying on this finding alone may lead to unnecessary interventions in children with transient synovitis. Muscle edema and bone marrow edema may support the diagnosis of septic arthritis. Clinical evaluation and inflammatory markers remain critical in guiding decisions for hip aspiration.

**Supplementary Information:**

The online version contains supplementary material available at 10.1007/s00256-025-05013-1.

## Introduction

Septic arthritis of the hip can lead to severe complications in children if left untreated, even for short periods. These complications include chronic femoral bone marrow edema, avascular necrosis of the femoral head, physeal damage, articular cartilage damage, acetabular dysplasia, hip instability, and limb-length discrepancy [1–5]. Consequently, septic hip arthritis is considered a medical emergency. However, in children aged < 10 years, the most common etiology of acute hip pain is transient synovitis, a self-limiting disorder [6]. Children have a 3% risk of experiencing transient synovitis at some point in their lives (accounting for 0.4–0.9% of pediatric emergency department visits), with variable recurrence rates [7]. In contrast, the incidence of septic hip arthritis in developed countries is 1–5 per 100,000 individuals [5].

Both septic arthritis and transient synovitis typically present with hip pain lasting1 to 33 days, often accompanied by limping or refusal to bear weight. This challenge is particularly evident in *Kingella kingae* osteoarticular infections, which often present indolently with moderate inflammatory marker elevation [8]. While transient synovitis can be managed non-operatively with supportive care and anti-inflammatory medication, septic arthritis requires early intervention with a multidisciplinary approach involving operative hip joint irrigation, debridement, and antibiotic treatment. Prompt diagnosis of septic arthritis is essential to facilitate early treatment and prevent long-term sequelae.

The gold standard for diagnosing septic hip arthritis is synovial fluid analysis, including cell count and culture. However, arthrocentesis is an invasive procedure that often requires anesthesia in young children. To identify patients who should undergo fluid sampling, Kocher et al. developed a predictive algorithm to differentiate transient synovitis from septic arthritis. This algorithm uses four clinical predictors, including a history of fever > 38.5 °C, refusal to weight bear on the affected extremity, erythrocyte sedimentation rate (ESR) > 40 mm/h, and serum white blood cell count (WBC) > 12,000/mm^3^ [9, 10]. Although the algorithm demonstrated strong initial and validated performance, subsequent studies have failed to replicate its discriminative accuracy [11]. Caird et al. proposed a modification by adding C-reactive protein (CRP) > 2.0 mg/L, given CRP’s strong association with septic arthritis of the hip [12]. However, this modified algorithm also struggled to discriminate septic hip arthritis from transient synovitis [11]. Therefore, the limited utility of current predictive algorithms, the invasiveness of diagnostic procedures, and the high morbidity associated with delayed treatment highlight the need for more reliable, non-invasive methods for diagnosing septic arthritis in children.

In addition to septic arthritis and transient synovitis, other etiologies with overlapping clinical presentations include transient synovitis, osteomyelitis, pyomyositis, Legg-Calvé-Perthes disease, juvenile idiopathic arthritis, slipped capital femoral epiphysis, Lyme disease, sickle cell anemia, and leukemia [5]. Magnetic resonance imaging (MRI) is highly sensitive for diagnosing these conditions and detecting hip effusion and soft tissue inflammation. In our practice, children with clinical and laboratory abnormalities suggestive of septic arthritis undergo MRI due to the strong association with concomitant bone marrow edema, which cannot be diagnosed through arthrocentesis [13]. Given its diagnostic value, MRI is a valuable tool for identifying the underlying cause of acute hip pain, particularly in patients with inconclusive clinical evaluations and borderline inflammatory indices. Prior studies suggested that decreased hip enhancement can differentiate between septic hip arthritis and transient synovitis [14, 15]. However, based on our experience, decreased femoral head enhancement is not specific to septic hip arthritis and can also occur in children with transient synovitis. This study evaluates the hypothesis that decreased femoral head enhancement on MRI does not allow a radiologist, blinded to clinical information, to reliably differentiate between septic hip arthritis and transient synovitis.

## Methods

This study complied with the Health Insurance Portability and Accountability Act and was approved by our institutional review board with a waiver of informed consent. We retrieved all children younger than 10 years who underwent intravenous contrast-enhanced MRI of the pelvis or hips between 2006 and 2023, with a report of hip joint effusion, from the radiology information system. We included children with symptoms lasting up to 2 weeks who were evaluated for musculoskeletal infectious processes in the pelvis or proximal thighs. From the electronic medical records, we retrieved demographic, clinical, laboratory, and pathological data, along with patients’ clinical courses and final diagnoses.

### Evaluation of the pelvis/hip MRI

Table S1 provides the details of the MRI protocol. Post-IV gadolinium injection coronal SET1 with fat suppression was performed, with acquisition times of 3:29 and 2:12 (minutes:seconds) on 1.5 T and 3.0 T MRI, respectively. The MRI studies were de-identified, anonymized, and independently reviewed by two pediatric radiologists with 2 and 7 years of post-fellowship experience, respectively. Both radiologists were blinded to clinical information and assessed the following findings:

- Bone marrow: normal, bone marrow edema, or other pathology.
- Periosteum: normal, periosteal reaction (a linear or curvilinear bright signal om water sensitive sequence along the cortex), or subperiosteal abscess (accumulation of fluid in the subperiosteal space).
- Muscle tissues: normal, edema, or abscess.
- Hip effusion: normal, grade 1 (minimal effusion), grade 2 (sufficient effusion surrounding the femoral head), or grade 3 (capsular recess distention) [16].
- Bilateral hip effusions: at least grade 2 in both hips.
- Synovial enhancement: normal or increased compared to contralateral side.
- Qualitative femoral head enhancement: present or decreased/absent enhancement of the femoral head ossification center or the femoral head cartilage in the absence of the ossification center.

Disagreements on qualitative femoral head enhancement were resolved by a consensus read.

Quantitative femoral head enhancement in the coronal series was assessed by a third pediatric radiologist with 17 years of post-fellowship experience. Drawing a freehand region of interest, the radiologist outlined each femoral head ossification center and recorded the average signal intensity. The difference in the average signal intensity between the femoral heads was calculated.

### Definitions

#### Definite septic arthritis

Defined by a positive joint fluid culture or a joint fluid WBC count ≥ 50,000 cells/µL with polymorphonuclear leukocyte predominance, along with a positive blood culture [17, 18].

#### Probable septic arthritis

Defined by a negative joint fluid culture, Gram stain, and blood cultures in patients without underlying conditions affecting the femoral head or hip joint (e.g., post-trauma, tumors, Legg-Calvé-Perthes disease, hip dysplasia, bone dysplasia, history of SCFE, or juvenile idiopathic arthritis) and presenting with the following characteristics:

1. Joint fluid WBC ≥ 50,000 cells/µL with polymorphonuclear leukocyte predominance [17, 18].
2. At least two of the following: fever ≥ 38.5 °C, ESR ≥ 40 mm/h, CRP ≥ 2.0 mg/dL, or WBC > 12,000 cells/µL [10, 12].

#### Transient synovitis

Defined by a negative joint fluid culture and Gram stain with a joint fluid WBC count < 50,000 cells/µL in children without adjacent bone marrow edema or pyomyositis and symptoms that decrease or resolve without antibiotic treatment.

#### Probable transient synovitis

Defined by no hip joint fluid obtained, without underlying conditions affecting the femoral head or hip joint (e.g., post-trauma, tumors, Legg-Calvé-Perthes disease, hip dysplasia, bone dysplasia, history of SCFE, or juvenile idiopathic arthritis) or other infections, no antibiotic treatment, and symptoms that decrease over time.

Kocher scores were determined retrospectively based on clinical and laboratory data.

### Statistical analysis

Study data were collected and managed using a research electronic data capture tool (REDCap, REDCap Consortium, Vanderbilt University Medical Center). Patient characteristics were compared between the two groups using a two-sample Student’s *t*-test for continuous variables and Fisher’s exact test for categorical variables. The sensitivity and specificity of septic arthritis diagnosis were calculated for the Kocher and modified Kocher scores, individual components of the Kocher scores, and decreased femoral head enhancement on MRI. A 5% significance level was used for all tests.

Cases of definite and probable septic arthritis were considered positive, whereas other etiologies were considered negative.

- True positive: Presence of definite or probable septic arthritis with positive MRI findings, as described above.
- False positive: Absence of definite or probable septic arthritis with positive MRI findings, as described above.
- True negative: Absence of definite or probable septic arthritis with negative MRI findings, as described above.
- False negative: Presence of definite or probable septic arthritis with negative MRI findings, as described above.

Inter-reader agreement between the two radiologists for assessing decreased femoral head enhancement was evaluated using the kappa statistic. Agreement was classified based on kappa coefficients as follows: none to slight (0.01–0.20); fair (0.21–0.40); moderate (0.41–0.60); substantial (0.61–0.80); almost perfect agreement (0.81–1.00).

## Results

### Patient selection

**Fig. 1:**
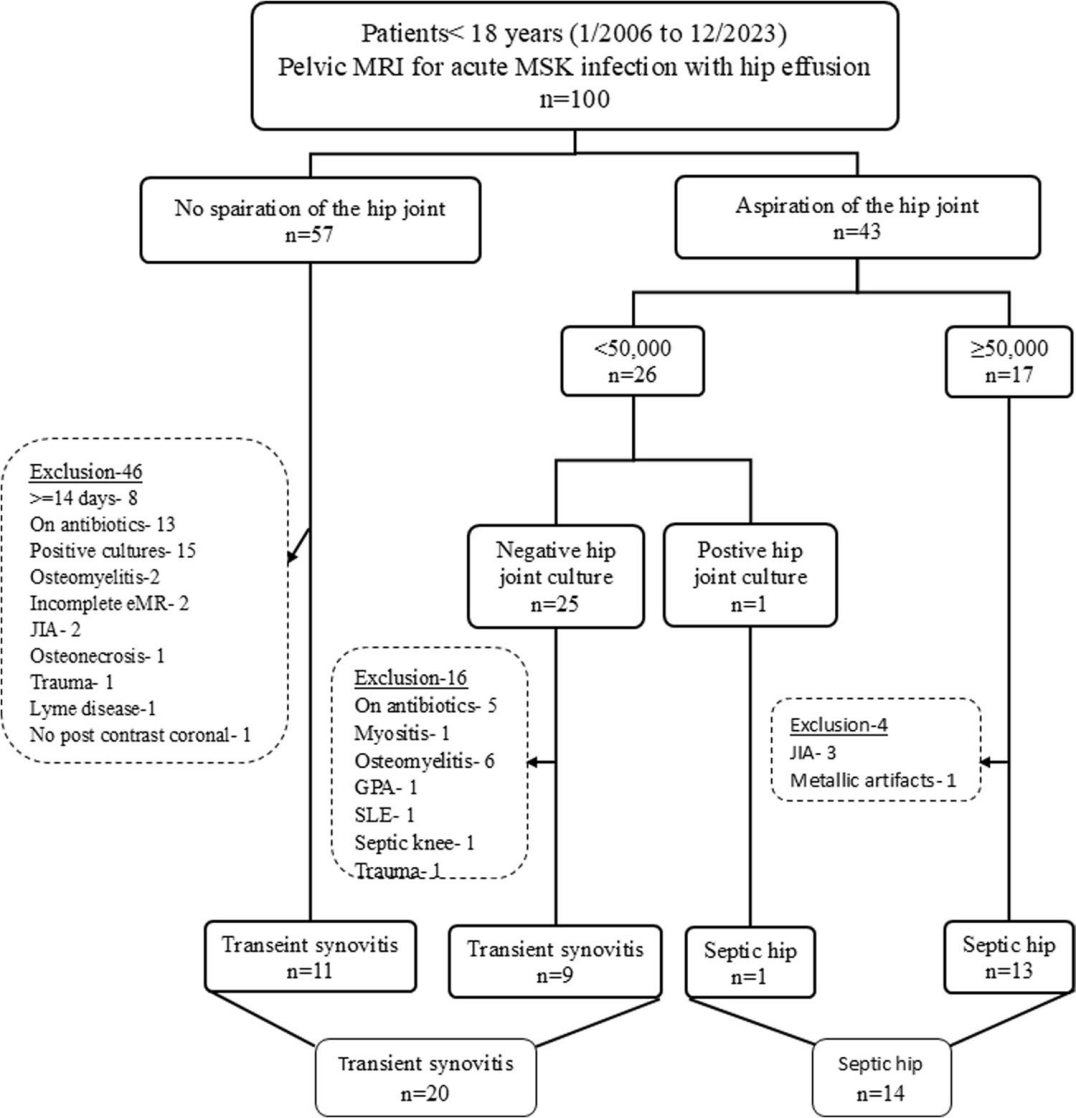
Flowchart of children with septic hip arthritis and transient synovitis; > 50,000 indicates white blood cells per milliliter in the aspirated synovial fluid. eMR, electronic medical records; JIA, juvenile idiopathic arthritis; GPA, granulomatosis with polyangiitis; SLE, systemic lupus erythematosus

Figure 1 presents the flowchart of the patient selection process. A total of 100 children underwent post-contrast pelvic MRI with reported hip effusion. Hip aspiration was not performed in 57 cases, of which 46 were excluded for the following reasons: positive cultures (*n* = 15; blood: *n* = 10, hip effusion: *n* = 4, muscle abscess: *n* = 1), prior antibiotic treatment (*n* = 13), symptoms lasting ≥ 14 days (*n* = 8), bone marrow edema (*n* = 2), juvenile idiopathic arthritis (*n* = 2), incomplete medical records (*n* = 2), femoral head osteonecrosis (*n* = 1), trauma (*n* = 1), Lyme disease (*n* = 1), and no post-contrast coronal SET1 FS imaging (*n* = 1). The remaining 11 patients were diagnosed with probable transient synovitis. Hip aspirations were performed in 43 children. Among them, 26 had a WBC count < 50,000 cells/µL. Of these, one had a positive hip joint culture, while 25 had negative cultures. Sixteen of these culture-negative cases were excluded for the following reasons: bone marrow edema (*n* = 6), prior antibiotic treatment (*n* = 5), underlying myositis (*n* = 1), septic knee (*n* = 1), trauma history (*n* = 1), granulomatosis with polyangiitis (*n* = 1), and systemic lupus erythematosus (*n* = 1). The remaining nine children were diagnosed with transient synovitis. In 17 children with hip aspiration, the WBC count was ≥ 50,000 cells/µL. Of these, four were excluded for juvenile idiopathic arthritis (*n* = 3) and metallic artifacts limiting the evaluation of femoral head enhancement (*n* = 1). The remaining 13 children were diagnosed with septic arthritis: three had positive cultures (definite septic arthritis), and 10 had negative hip joint cultures (probable septic arthritis).

Overall, 20 and 14 children were diagnosed with transient synovitis and septic hip arthritis, respectively.

### Patient characterization

Children with transient synovitis had a significantly higher male-to-female ratio (12:8) than those with septic arthritis (3:11; *p* = 0.038). Additionally, children with transient synovitis were significantly older (mean age: 6.05 ± 2.17 years) than those with septic arthritis (4.2 ± 2.78 years; *p* = 0.031) (Table 1).

**Table 1:** Demographic and clinical characteristics of 14 children with septic arthritis and 20 children with transient synovitis

	Septic hip; *n* = 14	Transient synovitis; *n* = 20	*P*-value
Gender			
Male	3 (21.4%)	12 (60%)	0.038
Female	11 (78.6%)	8 (40%)	
Age			
Range (years)	0.15–8.5	2.1–8.3	
Average (years) ± S.D	4.2 ± 2.78	6.1 ± 2.17	0.037
Location of the arthritis			0.080
Rt hip	10 (71.4%)	7 (35%)	
Lt hip	4 (28.6%)	12 (60%)	
Bilateral	(0%)	1 (5%)	
Cannot bear weight	12 (85.7%)	11 (55%)	0.076
Fever > 38.5 °C	6 (42.9%)	1 (5%)	0.012
WBC > 12 K cell/mL	7 (50%)	6 (30%)	0.296
ESR > 40 mm/h	6 (42.9%)	6 (30%)	0.495
CRP > 2 mg/dL	9 (64.3%)	10 (50%)	0.487
Kocher score (0–4)	2.21 ± 0.80	1.20 ± 0.95	0.003
Modified Kocher score (0–5)	2.86 ± 0.95	1.70 ± 1.30	0.008

Fever > 38.5 °C was significantly more prevalent in children with septic arthritis (6/14, 42.9%) than in those with transient synovitis (1/20, 5%; *p* = 0.012). No significant differences were found in arthritis lateralization, inability to bear weight, or any of the inflammatory indices. However, both the Kocher and modified Kocher scores were significantly higher in children with septic hip arthritis (*p* = 0.002 and 0.013, respectively). The mean Kocher score was 2.86 ± 0.95 in children with septic arthritis, compared to 1.70 ± 1.30 in those with transient synovitis. The mean modified Kocher score was 2.21 ± 0.80 in children with septic arthritis, compared to 1.20 ± 0.95 in those with transient synovitis. Using the Kocher score, 65% (13/20) of children with transient synovitis had a score of 0 or 1, compared to 14.3% (2/14) of children with septic arthritis. Using the modified Kocher score, 50% (10/20) of children with transient synovitis had a score of 0 or 1 (50%), whereas none of the children with septic arthritis had a score of 0 or 1.

Positive cultures were found in 35.7% (5/14) of patients with septic arthritis (35.7%), originating either from the hip effusion fluid (*n* = 3), blood (*n* = 1), or obturator externus abscess (*n* = 1). All cultures tested positive for *Staphylococcus aureus*.

### MRI findings

**Fig. 2:**
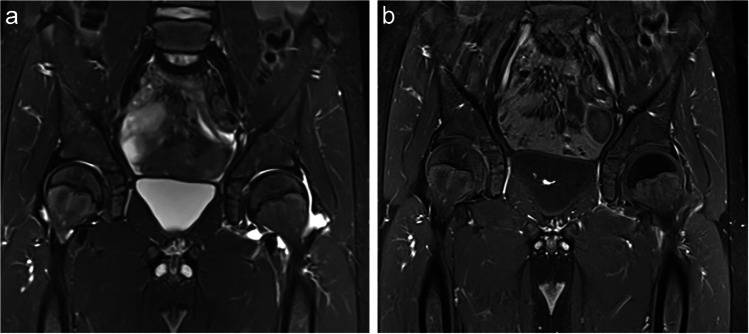
A 6-year-old boy with transient synovitis. The patient developed flu-like symptoms 10 days before admission and presented with 1 day of left hip pain, unable to bear weight on the day of admission. He was afebrile, with a white blood cell (WBC) count of 12,400 cells/mL, an erythrocyte sedimentation rate (ESR) of 43 mm/h, and a C-reactive protein (CRP) level of 3.5 mg/dL. Left hip aspiration yielded a WBC count of 1,150 cells/mL with a negative culture. He was treated with a non-steroidal anti-inflammatory drug (NSAID), resulting in symptom improvement. **A** coronal MRI STIR shows a large left hip effusion. **B** coronal MRI SET1 with fat suppression post-contrast shows decreased enhancement of the left femoral head

**Fig. 3:**
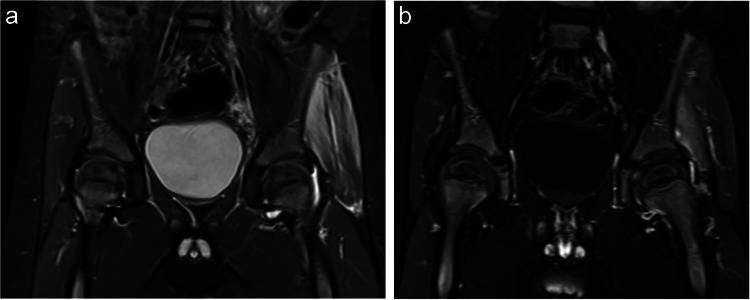
A 3-year-old boy with septic hip arthritis and myositis. The patient presented with a 3-day history of fever (38.9 °C) and refusal to bear weight. He had a normal white blood cell (WBC) count, an erythrocyte sedimentation rate (ESR) of 67 mm/h, and a C-reactive protein (CRP) level of 15 mg/dL. Left hip aspiration revealed a WBC count of 11,539 cells/mL, with a positive culture for *Staphylococcus aureus*. **A** coronal MRI STIR shows moderate left hip effusion and marked edema in the left gluteal muscles. **B** coronal MRI SET1 with fat suppression post-contrast shows normal enhancement of the left femoral head and enhancement of the left gluteal muscles

**Fig. 4:**
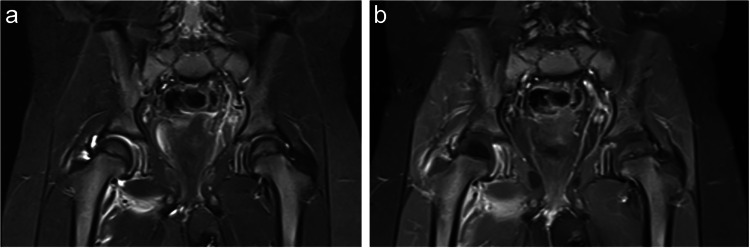
A 3-year-old girl with septic hip arthritis. The patient initially presented with 3 days of limping followed by a refusal to bear weight. She had a fever of 39.6 °C, a normal white blood cell (WBC) count, an erythrocyte sedimentation rate (ESR) of 80 mm/h, and a C-reactive protein (CRP) level of 4.3 mg/dL. Right hip aspiration revealed a WBC count of 175,000 cells/mL, with a positive culture for *Staphylococcus aureus*. **A** coronal MRI STIR shows moderate right hip effusion and edema in the adductor minimus muscle. **B** coronal MRI SET1 with fat suppression post-contrast shows decreased enhancement of the right femoral head

Table 2 summarizes the MRI findings. Most patients (78.6–100%) with septic arthritis and transient synovitis exhibited moderate-to-severe (grade 2 or 3) hip effusions. Muscle edema was significantly more common in patients with septic arthritis. Radiologist 1 identified muscle edema in 71.4% of septic arthritis cases, while Radiologist 2 identified it in 92.9% (Figs. 2 and 3) compared to 25.0% and 50.0% in transient synovitis cases, respectively (*p* = 0.013 and 0.011) (Fig. 4). Additionally, muscle abscesses were identified by one radiologist in two patients with septic arthritis. Both radiologists found only two patients with bone marrow edema, both of whom had septic arthritis.

**Table 2:** MRI findings in children with septic arthritis and transient synovitis

	Septic arthritis; *n* = 14		Transient synovitis; *n* = 20		*P*-values for radiologists 1 and 2
	Radiologist 1	Radiologist 2	Radiologist 1	Radiologist 2	
Muscle edema	10 (71.4%)	13 (92.9%)	5 (25.0%)	10 (50.0%)	0.013, 0.011
Muscle abscess	2 (14.3%)	0 (0%)	0 (0%)	0 (0%)	0.162, 1.00
Bone marrow edema	2 (14.3%)	2 (14.3%)	0 (0%)	0 (0%)	0.162, 0.162
Muscle edema or bone marrow edema	11 (84.6%)	13 (92.9%)	5 (25.0%)	10 (50.0%)	0.0013, 0.011
Effusion					
Mild	2 (14.3%)	0 (0%)	2 (10.0%)	0 (0%)	1.00, 1.00
Moderate/marked	11 (78.6%)	14 (100%)	18 (90.0%)	20 (100%)	0.627, 1.00
Bilateral					
Any grades	4 (28.6%)	10 (71.4%)	12 (60.0%)	10 (50.0%)	0.092, 0.296
Grades 2 or 3	2 (14.3%)	3 (21.4%)	4 (20.0%)	6 (30.0%)	0.672, 0.704
Decreased enhancement of the femoral head	11 (78.6%)	9 (64.3%)	9 (45.0%)	10 (50.0%)	0.079, 0.495
Decreased enhancement of the femoral head—consensus	10 (71.4%)		10 (50%)		0.296
Signal intensity difference between the femoral heads	59.1 ± 80.2		32.8 ± 29		0.258

Only one 53-day-old patient did not have yet a femoral head ossification center. Substantial agreement was found between the two radiologists for the overall determination of decreased femoral head enhancement (kappa = 0.70). Decreased femoral enhancement was commonly observed in patients with septic arthritis and transient synovitis, occurring in 78.6% and 64.3% of septic arthritis cases, and in 45.0% and 50.0% of transient synovitis cases, as assessed by Radiologists 1 and 2, respectively (*p* = 0.079 and *p* = 0.495). Based on a consensus read, decreased femoral enhancement was observed in 71.4% of patients with septic arthritis and in 50.0% of those with transient synovitis (*p* = 0.296). The signal intensity difference between the right and left femoral heads was also not significantly different between patients with septic arthritis (59.1 ± 80.2) and those with transient synovitis (32.8 ± 29.2; *p* = 0.258).

### Sensitivity and specificity of risk factors for septic hip arthritis

Table 3 summarizes the sensitivity and specificity of each risk factor included in the Kocher and modified Kocher scores, as well as the MRI findings. As expected, higher Kocher and modified Kocher scores were associated with increased specificity but decreased sensitivity. Decreased femoral head enhancement demonstrated moderate sensitivity (71.4%) but low specificity (50.0%). MRI-detected muscle edema had moderate-to-high sensitivity (71.4% and 92.9%) but moderate-to-low specificity (75.0% and 50.0%) for diagnosing septic hip arthritis, as assessed by Radiologists 1 and 2, respectively. Only one radiologist diagnosed bone marrow edema without muscle edema, increasing sensitivity to 78.6% without affecting specificity.

**Table 3:** Sensitivity and specificity of risk factors for septic hip arthritis

Risk factor	Sensitivity	Specificity
Cannot bear weight	85.7%	45.0%
Fever > 38.5	42.9%	95.0%
WBC > 12 K cell/mL	50.0%	70.0%
ESR > 40 mm/h	42.9%	70.0%
CRP > 2 mg/dL	64.3%	50.0%
Kocher score		
0 vs 1–4	100%	25.0%
0–1 vs 2–4	85.7%	65.0%
0–2 vs 3–4	28.6%	90.0%
0–3 vs 4	7.1%	100%
Modified Kocher score		
0 vs 1–5	100%	20.0%
0–1 vs 2–5	100%	50.0%
0–2 vs 3–5	57.1%	70.0%
0–3 vs 4–5	21.4%	90.0%
0–4 vs 5	7.1%	100%
Decreased femoral head enhancement	71.4%	50.0%
Muscle edema		
Radiologist 1	71.4%	75.0%
Radiologist 2	92.9%	50.0%
Muscle edema or osteomyelitis		
Radiologist 1	78.6%	75.0%
Radiologist 2	92.9%	50.0%

## Discussion

Our study differs from prior studies suggesting that femoral head perfusion on MRI can distinguish transient synovitis from septic hip arthritis [14, 15, 19–22]. We found that only muscle edema and bone marrow edema were associated with septic hip arthritis, whereas decreased femoral head enhancement was commonly seen in both conditions. Two previous studies proposed that decreased femoral head enhancement on MRI could help differentiate septic hip arthritis from transient synovitis [14, 15]. These studies included small patient cohorts, with a 2007 study including 11 patients with transient synovitis and nine with septic arthritis [14] and a 2012 study including 11 and seven patients, respectively [15]. MRI may be more selectively used to evaluate septic hip arthritis in these studies, resulting in a higher positive predictive value. Notably, in those studies, 89% [14] and 85.7% [15] of children with septic hip arthritis had decreased femoral head enhancement, whereas in our study, in a consensus read, only 71.4% of children with septic hip arthritis had decreased femoral head enhancement. Moreover, the specificity of decreased hip enhancement for diagnosing septic hip arthritis in our study was only 50%. In addition, our quantitative assessment did not show any significant difference between the two groups of patients.

The acquisition times for post-contrast enhancement of the femoral head in our study (3:29 and 2:12 min:seconds) fall within the reported time range (2:20–4:35 min:seconds) of maximal enhancement differentiation [15].

The etiology of decreased femoral head enhancement is likely related to increased intracapsular pressure, given the observed increase in intracapsular pressure and decrease in blood flow to the femoral head, as shown in a previous study on transient synovitis [23].

A few studies have suggested that MRI can differentiate septic hip arthritis from transient synovitis based on the presence of bone marrow and soft tissue edema in patients with septic hip arthritis [14, 15, 19–22]. In our study, only two patients had bone marrow edema, both of whom had septic arthritis. Both radiologists observed that muscle edema was significantly more common in patients with septic hip arthritis. The sensitivity of MRI findings of muscle edema alone for diagnosing septic hip arthritis was moderate to high (71.4% and 92.9%), whereas specificity was moderate to low (75.0% and 50.0%) for Radiologists 1 and 2, respectively. Bone marrow edema was diagnosed in two patients, both of whom had septic hip arthritis.

The primary evaluation of children with suspected septic hip relies on the combination of clinical evaluation and inflammatory indices. The Kocher scoring criteria [10] and the modified Kocher scoring criteria (Caird scoring) [12] are the most extensively evaluated clinical algorithms. However, several prospective studies have reported reduced sensitivity and specificity [9, 24]. In our study, the combination of risk factors from the Kocher and modified Kocher scoring criteria differed significantly between transient synovitis and septic hip arthritis. The modified Kocher scoring (0–1 vs. 2–5) provided the best differentiation, with a sensitivity of 100% and specificity of 50%.

Our study has several limitations. Although it includes a larger sample size than previous studies, the number of patients with septic arthritis and transient synovitis remains relatively small and lacks the statistical power to determine whether the difference in the prevalence of decreased femoral head enhancement between the two groups is significant. However, this limitation does not alter our main finding: decreased femoral head enhancement is common in both septic arthritis (71.4%) and transient synovitis (50%) and has a low specificity (50%) for distinguishing between the two conditions. In addition, it was a retrospective study. As a result, 11 children with transient synovitis did not undergo joint aspiration, and their diagnosis was based on clinical recovery without antibiotic treatment. While this approach reflects standard clinical practice, the possibility of indolent septic hip arthritis cannot be completely excluded, as most children with transient synovitis are diagnosed based on clinical criteria. Additionally, cultures were positive in only five of the 14 children with septic hip arthritis (35.7%), a rate consistent with previously reported ranges [5, 12, 25]. However, in culture-negative cases, alternative etiologies such as transient synovitis cannot be completely excluded. Second, MRI evaluation of children with transient synovitis introduces a selection bias, as it is more likely to be performed in those with more severe symptoms and potentially higher intracapsular pressure. Consequently, our results may not be generalizable to children with milder symptoms or those with negative or only mildly elevated levels of inflammatory markers. Finally, post-contrast MRI was not performed using a dynamic post-contrast technique, likely resulting in greater variability in the post-contrast scan delay time.

## Conclusion

Our findings indicate that decreased femoral enhancement on MRI is commonly observed in both transient synovitis and septic arthritis with poor sensitivity and specificity. Muscle edema and bone marrow edema are more commonly observed in children with septic hip arthritis, with varied specificities. Therefore, the decision to aspirate the hip joint should not be made based on MRI findings alone but should primarily rely on clinical evaluation and inflammatory indices.

## Supplementary Information

Below is the link to the electronic supplementary material.

Supplementary file1 (DOCX 18 KB)

## Data Availability

The data that support the findings of this study are available from the corresponding author upon reasonable request. Due to the nature of clinical data and patient privacy regulations, the data are not publicly available.
