# Supplementary material for: Can decreased femoral head enhancement differentiate between septic hip arthritis and transient synovitis?
Source: Skeletal Radiol. 2025 Aug 12;55(1):181–90. doi: 10.1007/s00256-025-05013-1 (PMC12627202; doi:10.1007/s00256-025-05013-1)
Supplement: Supplementary file 1 — Supplementary file1 (DOCX 18 KB) [file 256_2025_5013_MOESM1_ESM.docx]

**Table S1**─Pelvis MRI protocol

| **Sequence** | **TR**  **(ms)** | **TE**  **(ms)** | **Flip**  **Angle** | **NEX** | **Matrix** | **Slice Thickness (mm)** | **Gap (mm)** | **Acquisition**  **Time (minutes: seconds)** |
| --- | --- | --- | --- | --- | --- | --- | --- | --- |
| 1.5 T |  |  |  |  |  |  |  |  |
| Coronal T1W TSE | 572 | 13 | 150 | 1 | 384x269 | 5 | 1 | 2:07 |
| Coronal STIR | 3370 | 35 | 180 | 1 | 320X240 | 5 | 1 | 4:55 |
| Axial T1W TSE | 677 | 11 | 150 | 1 | 384X234 | 5 | 1 | 2:10 |
| Axial T2W TSE FS | 3500 | 86 | 180 | 1 | 256X232 | 5 | 1 | 2:50 |
| Sagittal T2 FS TSE | 6000 | 103 | 180 | 2 | 256X156 | 4 | 0.8 | 3:38 |
| Sagittal T1W TSE | 587 | 11 | 150 | 2 | 320X208 | 4 | 0.8 | 2:31 |
| Coronal T1W FS TSE + Contrast | 700 | 13 | 150 | 1 | 384X268 | 5 | 1 | 3:29 |
| Axial T1W FS TSE+ Contrast | 580 | 11 | 150 | 1 | 384X234 | 5 | 1 | 3:44 |
| 3 T |  |  |  |  |  |  |  |  |
| Coronal T1W TSE | 500 | 8.7 | 150 | 2 | 320X203 | 3 | 0.6 | 1:47 |
| Coronal STIR | 4280 | 39 | 130 | 2 | 320X195 | 3 | 0.6 | 2:57 |
| Axial T1W TSE | 721 | 12 | 120 | 2 | 320X144 | 4 | 0.8 | 1:47 |
| Axial T2W TSE FS | 4850 | 48 | 150 | 2 | 256X154 | 4 | 0.8 | 2:12 |
| Sagittal T2 FS TSE | 5170 | 48 | 150 | 2 | 320X208 | 5 | 1 | 2:16 |
| Sagittal T1W TSE | 541 | 12 | 120 | 1 | 320X256 | 5 | 1 | 1:47 |
| Coronal T1W FS TSE+ Contrast | 622 | 8.7 | 150 | 1 | 320X203 | 3 | 0.6 | 2:12 |
| Axial T1W FS TSE+ Contrast | 716 | 12 | 120 | 2 | 320x144 | 4 | 0.8 | 2:22 |

TSE = turbo spin echo

FS= fat suppression
